# An Optimized Handover Scheme with Movement Trend Awareness for Body Sensor Networks

**DOI:** 10.3390/s130607308

**Published:** 2013-06-03

**Authors:** Wen Sun, Zhiqiang Zhang, Lianying Ji, Wai-Choong Wong

**Affiliations:** 1 Department of Electrical and Computer Engineering, National University of Singapore, Singapore 119613, Singapore; E-Mail: wong lawrence@nus.edu.sg; 2 Department of Computing, Imperial College, London SW7 2AZ, UK; E-Mail: z.zhang@imperial.ac.uk; 3 Graduate University of Chinese Academy of Sciences, Beijing 100049, China; E-Mail: jilianying@gucas.ac.cn

**Keywords:** localization, handover, body sensor network

## Abstract

When a body sensor network (BSN) that is linked to the backbone via a wireless network interface moves from one coverage zone to another, a handover is required to maintain network connectivity. This paper presents an optimized handover scheme with movement trend awareness for BSNs. The proposed scheme predicts the future position of a BSN user using the movement trend extracted from the historical position, and adjusts the handover decision accordingly. Handover initiation time is optimized when the unnecessary handover rate is estimated to meet the requirement and the outage probability is minimized. The proposed handover scheme is simulated in a BSN deployment area in a hospital environment in UK. Simulation results show that the proposed scheme reduces the outage probability by 22% as compared with the existing hysteresis-based handover scheme under the constraint of acceptable handover rate.

## 1. Introduction

A body sensor network (BSN) enables wireless communications between several miniaturized body sensors and a single coordinator worn on the human body. Since a BSN allows continuous health monitoring with real-time updates of medical records through Internet, it plays a crucial role in the next generation healthcare technologies [1–5]. When a BSN that is linked to the Internet via a wireless local area network (WLAN) interface moves from one coverage zone to another, a handover is required to maintain network connectivity. There are two parameters associated with a handover decision: handover rate and outage probability. The higher the handover rate, the more network resources would be consumed to reroute the communication from one interface to another. However, when the handover rate is low, handover may not be performed promptly, which causes signal-to-noise ratio (SNR) below an acceptable threshold, called outage. In BSNs where physiological information is transmitted with limited network resources, both handover rate and outage should be strictly reduced [6,7].

Conventional handover approaches have been mostly developed by cellular network researchers. The received signal strength indicator (RSSI) is commonly used as a metric [8,9], where the RSSI from the serving base station (BS) is compared with that from a target BS, and decisions are made using a constant margin. However, the fluctuations of RSSI associated with shadow fading cause a call to be repeatedly handed over back and forth between neighboring BSs, referred to as unnecessary handover [8,9]. To suppress the unnecessary handover, several location-based handover algorithms have been proposed using timers or hysteresis [10–13]. Most studies assume that the location of the mobile can be determined using the global positioning system (GPS), but GPS does not fit well in indoor environments where BSNs are commonly deployed [10]. Non-GPS-based solutions for indoor localization have been developed using RSSI-based WLAN localization or sensor-based tracking. WLAN RSSI-based tracking scheme [14,15] estimates the distance between the target and reference points using the path loss function between RSSI and distance. Unfortunately, in indoor environments, the wireless channel is very noisy and radio frequency (RF) signal may suffer from reflection, diffraction and multipath effect, which makes the RSSI a complex function of distance. To overcome this problem, the WLAN fingerprinting scheme uses a priori radio map to capture the RSSI of each access point (AP) at certain points in the area of interest and live RSSI values are then compared with the radio map to find the closest match [11]. The major disadvantages of fingerprinting tracking scheme are the needs for dense training coverage and poor extrapolation to areas not covered during training. Another alternative indoor localization is inertial sensor-based kinematic tracking, which adopts kinematic relationship to estimate pedestrian localization [16]. However, the kinematic tracking is subject to the accumulated measurement errors. In this paper, we compensate the differences of the kinematic tracking and the WLAN RSSI-based tracking to get more accurate position estimation of a BSN user, based on which handover decision is made.

Note that handover performance is related not only with the current position of a BSN but also with the movement trend (movement pattern, direction, and velocity) of the BSN user [17]. In this paper, we propose an optimized handover scheme with movement trend awareness for BSNs. The proposed scheme predicts the future position of a BSN user using the movement trend extracted from historical positions, and adjusts the handover decision accordingly. Furthermore, the geometric constraints are utilized for the position prediction when the floor map of the BSN deployment area is known.

The main contributions of this paper are as follows:

- A relatively accurate trajectory of BSN is obtained by fusion of WLAN RSSI-based tracking and inertial sensor-based kinematic tracking, which can be easily obtained in BSNs.
- A real-time user profile is built for each BSN to indicate the movement trend of the BSN.
- Handover initiation time is optimized when the unnecessary handover rate is estimated to meet the requirement and the outage probability is minimized.

The remainder of this paper is organized as follows. Section 2 describes the network model. Section 3 introduces the design of the proposed handover scheme. Then Section 4 compares the simulation results of the proposed handover scheme with the existing handover schemes in two scenarios. Finally, Section 5 concludes this paper.

## 2. Network Model

**Figure 1.**
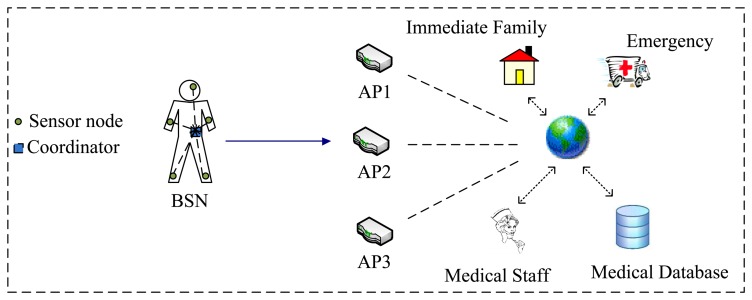
The common BSN architecture.

Figure 1 illustrates the common architecture of a BSN. The physiological information collected by sensor nodes is first delivered to a coordinator on the body, which then forwards the information to the concerned agents via a WLAN access point [18–20]. In this paper, we only consider the communication between the coordinator and the AP for handover analysis.

We assume that BSNs are equipped with inertial sensors such as accelerator, gyroscope, and magnetometer for healthcare applications. The raw data from the accelerator is first filtered with a low-pass filter at 5 Hz. Then we decouple the linear acceleration in the geocentric coordinate system using the results from the gyroscope and magnetometer, as the measured acceleration is the combination of gravity and linear acceleration. For details please refer to [16]. Based on linear acceleration, kinematic tracking scheme adopts kinematic relationships to estimate the position of a BSN.

In addition, we assume that multiple APs (at least three) are placed with overlapping coverage within the area of interest. A BSN measures its RSSI from these APs, which are being used as reference points, and calculate the distances by the Log distance path loss model. A typical example of WLAN based tracking includes the Horus localization system [21].

In this paper, we compensate the differences of the kinematic tracking scheme and the WLAN RSSI-based scheme, and fuse them to get a more accurate position prediction.

## 3. Proposed Handover Scheme

The proposed handover scheme has the following modules: (1) position tracking; (2) position prediction; and (3) handover decision. In this section, we first give an overview of the proposed scheme and then elaborate the three component modules.

### 3.1. Overview of the Proposed Handover Scheme

The proposed handover scheme comprises the following steps:

- **Step 1** (Position tracking): The ***position tracking*** (see Section 3.2) is initiated when the RSSI from the current AP falls below a certain threshold.
- **Step 2** (Position prediction): When the BSN user crosses the AP boundary, ***position prediction*** (see Section 3.3) is made based on the movement trend extracted from position tracking.
- **Step 3** (Handover decision): The coordinator of the BSN executes the ***handover decision*** (see Section 3.4) to determine the handover initiation time.
- **Step 4** (Handover initiation): Handover is performed at the handover initiation time when the actual position of the BSN is within an acceptable deviation range from the predicted position. Otherwise, handover is performed immediately.

**Figure 2.**
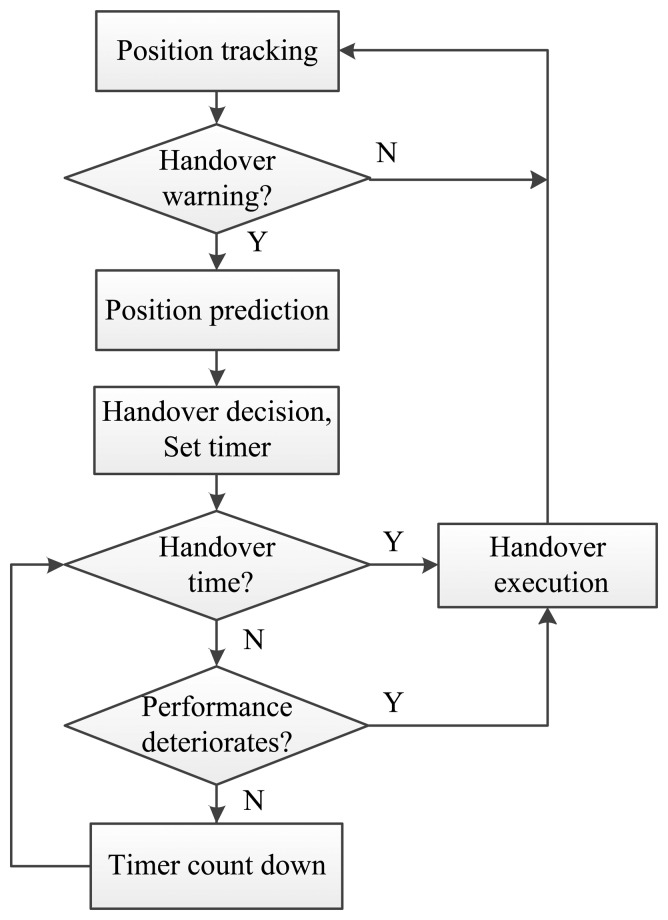
Flow chart of the proposed handover scheme.

The overall flow chart of our proposed approach is shown as Figure 2.

### 3.2. Position Tracking

In Step 1 of the workflow described in Section 3.1, the position tracking is executed when the RSSI from the current AP decreases significantly. The position of the BSN user is obtained by fusion of inertial sensor-based kinematic tracking scheme and WLAN RSSI-based tracking scheme. A Kalman filter [22] is used as the fusion tool to compensate the differences of the two tracking scheme and improve the positioning accuracy.

The state vector of the Kalman filter is expressed as *X_k_* = [*s*, *v*, *a*]^*T*^, where *s*, *v*, *a* are the target position, velocity, and acceleration respectively. Each of them is a two-dimensional vector along the x-axis and y-axis, *i.e.*, *s* = [*s_x_*, *s_y_*]^*T*^, *v* = [*v_x_*, *v_y_*]^*T*^, *a* = [*a_x_*, *a_y_*]^*T*^. The system state transition function of the filter can be expressed as

(1) $$X_{k}=F_{k}X_{k-1}+w_{k}$$

where *F_k_* is the state transition matrix and *w_k_* is the process noise. We utilize the Wiener-process acceleration model (WPAM) [23], where the acceleration is a Wiener process and *F_k_* = [1, *T*, *T*^2^/2; 0, 1, T; 0, 0, 1]. This system state transition function performs the kinematic tracking.

In the measurement vector *z_k_* = [*s*, *a*]*^T^*, *s* is obtained by the WLAN RSSI-based Horus system [21], while a is calculated based on readings from the inertial sensors [24]. The observation equation is

(2) $$z_{k}=H_{k}X_{k}+n_{k}$$

where *H_k_* is the observation matrix and *n_k_* is the measurement noise that is determined empirically.

From the output of the Kalman filter, a relatively accurate trajectory of the BSN user is achieved. In addition, it provides the real-time velocity information, which is to be utilized in position prediction.

### 3.3. Position Prediction

In Step 1 of the workflow described in Section 3.1, the position prediction is performed when the BSN crosses the AP boundary. Note that the future trajectory of a BSN would possibly follow the similar movement trend with its historical trajectory. In this study, we utilize the average velocity *v̅* over the last *N* time slots of the historical trajectory as the movement trend to predict future position.

To model the future positions, we made the following assumptions:

- The future trajectory of a BSN follows the WPAN model [23];
- The future trajectory starts with the velocity of *v̅*;
- The future acceleration ai follows a white Gaussian distribution, *i.e.*, $a_{i}∼N(0,\sigma_{a}^{2})$, where $\sigma_{a}^{2}$ is extracted from the historical trajectory by taking their variances, *i.e.*, $\sigma_{a}^{2}=\mathrm{var}[a_{k-1},a_{k-2},\ldots ,a_{k-N}]$.

The movement trend *v̅* is expressed as

(3) $$\bar{v}=\frac{v_{k-1}+v_{k-2}+\ldots +v_{k-n}}{N},k>N$$

where [*v*_*k*−1_, *v*_*k*−2_, … *v_k−N_*] is the set of historical velocities, and *k* is the current time index. According to the movement trend, a series of future positions (*s_i_*, *k* < *i* ≤ *k* + *τ*) is predicted as follows:

(4) $$v_{i}=\{\begin{array}{c} \bar{v},i=k\\ v_{i-1}+a_{i}T,k<i\leq k+\tau \end{array}$$

(5) $$s_{i}=s_{k}+\sum_{j=k}^{i}\left(\frac{v_{j-1}+v_{j}}{2}\right)T=s_{k}+(i-k)T\bar{v}+\sum_{j=k}^{i}T\left[(i-j)+\frac{1}{2}\right]a_{j}$$

(6) $${\overset{⌢}{s}}_{i}=E[s_{i}]=s_{k}+(i-k)T\bar{v}$$

The notations involved are summarized in Table 1. The future velocity *v_i_* at the time index *i* is predicted based on the starting velocity of *v̅* and historical acceleration *a_i_*. Assuming that *a_i_* follows Gaussian distribution, the predicted position *s_i_* also follows a Gaussian distribution with a mean of *ŝi* and an accumulated variance.

**Figure 3.**
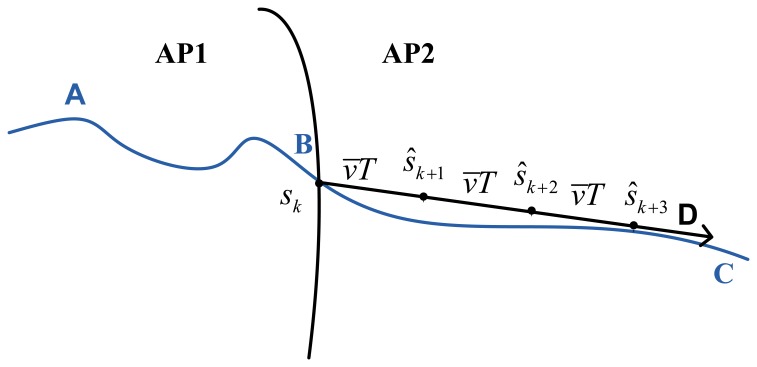
Position prediction.

Figure 3 depicts the procedure of position prediction. As can be seen, curve *AB* is the historical trajectory of the BSN. When the BSN crosses the AP boundary at point *B* (also denote as *s_k_*), position prediction is performed, and *ŝ_k_*, *ŝ*_*k*+1_, and *ŝ*_*k*+2_ are the predicted positions in the time index *k*, (*k* + 1), and (*k* + 2) respectively.

### 3.4. Handover Decision

Based on the position prediction, handover initiation time is determined when the unnecessary handover rate is estimated to meet the requirement and the outage probability is minimized.

#### 3.4.1. Confidence Probability

In order to estimate the unnecessary handover probability, we define the confidence probability Pr*_con_*(*i*) as the probability that at time index *i* a BSN actually enters the coverage of the target AP, conditioned on the BSN being predicted to be within the coverage of the target AP. As such, the unnecessary handover probability, which is the probability that the BSN would be back to the previous AP at the time index *i*, can be expressed as (1 − Pr*_con_*(*i*)).

**Figure 4.**
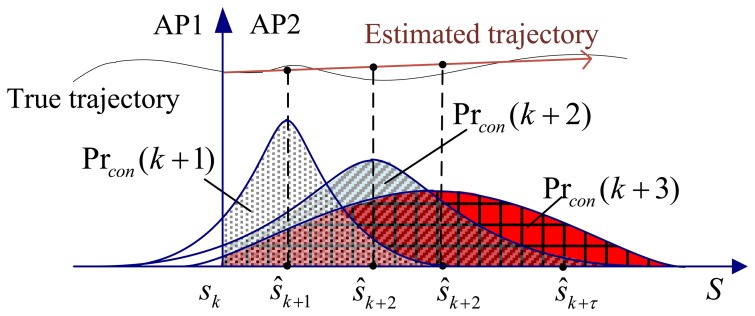
Confidence probability prediction.

To calculate Pr*_con_*(*i*), we map the predicted position *s_i_* along the orthogonal and parallel directions of the AP boundary, as shown in Figure 4. Denote its orthogonal mean and standard deviation as Δ*s̃_i_* and *σ̃_i_* respectively. Note that only the orthogonal factors contribute to the handover decision. The confidence probability is expressed as

(7) $${\text{Pr}}_{\text{con}}(i)=1-Q\left(\frac{\Delta {\tilde{s}}_{i}}{{\tilde{\sigma }}_{i}}\right)$$

where *Q*(·) is the complementary distribution function of the standard Gaussian [25]. Denote *P* as the mapping vector that is orthogonal to the AP boundary. Δ*s̃_i_* and *σ_i_* are calculated as follows:

(8) $$\Delta {\tilde{s}}_{i}=({\hat{s}}_{i}-s_{k})P=(i-k)T\bar{v}P$$

(9) $${\tilde{\sigma }}_{i}^{2}=E\left[P^{T}{\left(s_{i}-E\left[s_{i}\right]\right)}^{2}P\right]=P^{T}\sigma_{s_{k}}^{2}P+\frac{{\left(i-k\right)}^{2}T^{2}}{4}P^{T}\sigma_{a}^{2}P$$

where $\sigma_{s_{k}}^{2}$is the uncertainty of the current point *s_k_*.

As can be observed in Equation (7), Δ*s̃_i_*/*σ̃_i_* increases with *i*, thus Pr*_con_*(*i*) also increases with *i*. It means that the farther the predicted position of the user is from the boundary of the serving AP, the higher the probability that the user is actually within the coverage of the target AP. In Figure 4, Pr*_con_*(*i*) is the shadow part under the probability density distribution (PDF) of *s_i_* at AP2 side. Pr*_con_*(*k*+1), Pr*_con_*(*k*+2), Pr*_con_*(*k*+3) are the confidence probability at the time index *k*, (*k* + 1), and (*k* + 2) respectively.

#### 3.4.2. Handover Initiation

Based on the confidence probability, we determine the optimized handover initiation time. This is a tradeoff problem. The higher the handover rate, the more network resources would be consumed to reroute the communication. However, when the handover rate is low, handover may not be performed promptly resulting in outage. Thus the determination of handover time should meet the unnecessary handover probability requirement and the outage probability requirement concurrently.

A handover from AP1 to AP2 occurs at time index *i* when the following two criteria are satisfied,

(10) $$\text{Criterion}1:1-{\text{Pr}}_{\text{con}}(i)\leq \alpha$$

(11) $$\text{Criterion}2:{\text{Pr}}_{\text{con}}(i)\leq \beta$$

where *α* is the required unnecessary handover rate and *β* is the tolerable outage probability. Criterion 1 is to ensure the unnecessary handover rate requirement, while Criterion 2 is to ensure the outage probability requirement.

**Figure 5.**
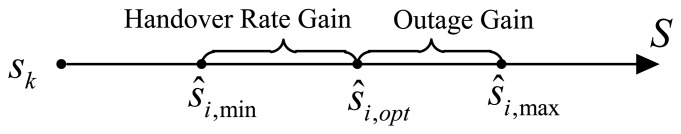
Performance gain.

As shown in Figure 5, *ŝ*_*i*,min_ is the point where Criterion 1 is met, *ŝ*_*i*,max_ is the point where the Criterion 2 is met. Thus any *i*_*opt*_ ∈ [*i*_min_, *i*_max_] satisfies the two criteria concurrently. If *i_pot_* is chosen closer to *i*_min_, the outage probability could be further reduced, which is shown as outage gain. Conversely, if *i_pot_* is closer to *i*_max_, the handover rate could be further reduced, which is shown as handover rate gain. As outage is crucial for BSNs, we choose the point *i_opt_* = *i*_min_ as the optimal handover initiation time, when the unnecessary handover rate is estimated to meet the requirement and the outage probability is minimized.

## 4. Simulation Results and Discussion

We implemented the proposed handover scheme in the MATLAB 7.0 simulator. We first compare the proposed localization scheme with the WLAN RSSI-based scheme. Then the proposed handover scheme is considered in two scenarios: a BSN deployment scenario without constructive constraints and a realistic BSN deployment scenario with constructive constraints.

### 4.1. Simulation Settings

The BSN deployment area is covered by the WLAN, commonly based on an IEEE 802.11a/b/g/n network. We assume that APs of WLAN follow traditional hexagonal layout, and the coverage radius of each AP is 50 meters. BSNs move inside the BSN deployment area following WPAN model. The pedestrian characteristics of each BSN user are set according to [26]. The sensor radio of a BSN has a 1024 Kbps data rate and outage SNR of −10 dB. To remove the effect of differing initial conditions on the performance, we run the simulation fifty times with different initial BSN positions and then calculate the average results. In the simulation, the position of a BSN is predicted through the experimental results from WLAN RSSI-based Horus system [21] and inertial sensor-based kinematic tracking [27].

The performance of the proposed handover is compared with the basic scheme and the hysteresis-based handover. The basic scheme [9] performs handover once the BSN crosses the AP boundary, while the hysteresis-based handover scheme performs handover until the hysteresis requirement is met.

### 4.2. Simulation Results of the Localization Scheme

**Figure 6.**
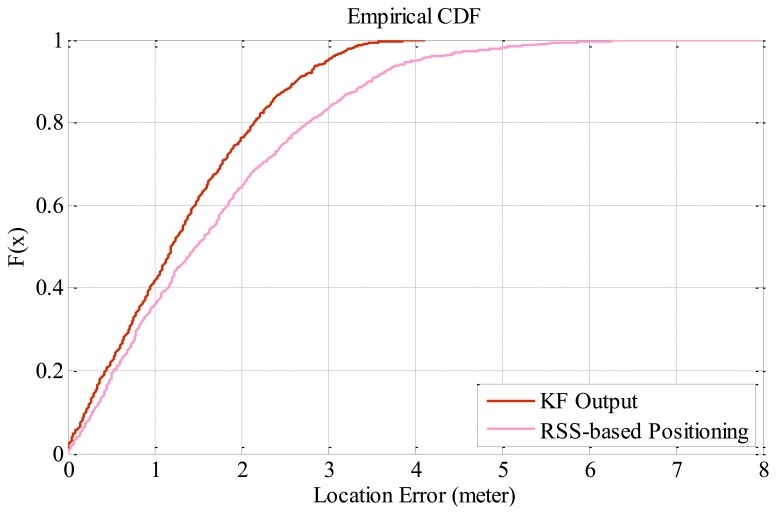
Empirical CDF comparison of Kalman filter output and RSSI-based tracking.

Figure 6 compares the cumulative distribution function (CDF) of location errors using the proposed tracking approach and WLAN RSSI-based tracking. As expected, the positioning accuracy achieves significant improvement by fusion of the kinematic tracking and WLAN triangular tracking. In particular, 90% of the location errors are within 2.5 m, while that of RSSI-based tracking stays within 3.5 meters.

Table 2 investigates the performance of the proposed handover scheme over various localization errors. It can be seen that within tolerable error range the proposed handover performance changes slightly with location errors. This is because the proposed scheme is mainly based on the movement trend and user profile, which is extracted from a large volume of historical data.

### 4.3. Simulation Results in the Case without Constructive Constraint

**Figure 7.**
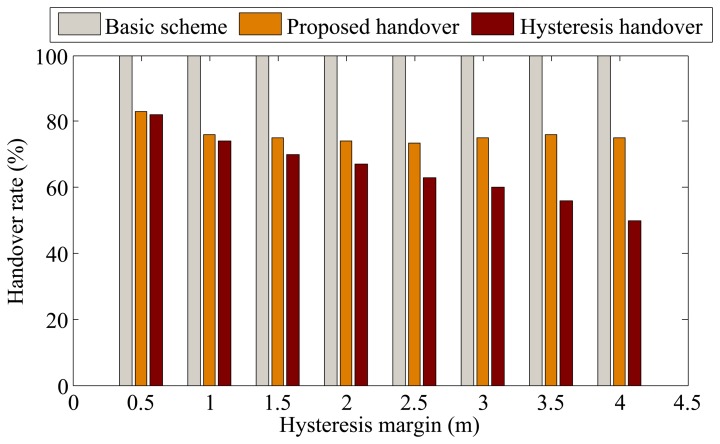
Comparison of handover rate in the BSN deployment case without constructive constraints.

**Figure 8.**
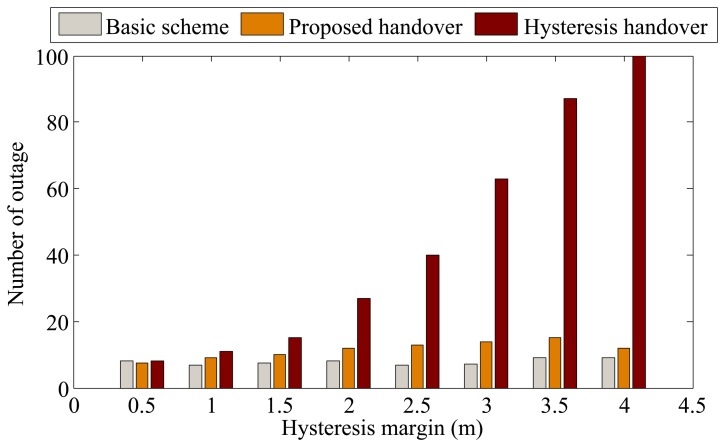
Comparison of the number of outage in the BSN deployment case without constructive constraints.

In this part, we consider the BSN deployment case without constructive constraints, where BSNs move inside an area of 100 × 100 square meters following the mobility model of WPAM [23,28]. The handover performance is investigated under different hysteresis margins. For the proposed handover method, hysteresis margin refers to the distance between the APs boundary and the position where the outage requirement is met. By changing the hysteresis margin, the outage requirement is also changed. The results are shown in Figures 7 and 8.

Instead of showing the actual number of handovers performed for the three schemes, Figure 7 presents the number of handovers normalized by the number of handovers from the basic scheme. As expected, the handover rate of the basic scheme is the highest, as handover is implemented each time the BSN crosses the AP boundary. The handover rate of the hysteresis-based scheme decreases as the hysteresis margin increases. For the proposed scheme, the handover rate also decreases and remains similar to that of the hysteresis-based scheme when the hysteresis margin is relatively small. This is because a BSN is more likely to avoid unnecessary handover when the hysteresis margin increases. When the hysteresis margin increases, the handover rate of the proposed scheme remains stable since most of the unnecessary handovers have been alleviated.

Figure 8 shows the comparison of the number of outages for the three schemes. As can be seen, the number of outage of the basic scheme is lowest at the expense of much higher handover rate as shown in Figure 7. The number of outages of the proposed scheme significantly outperforms that of hysteresis-based handover (about 22% on average), especially when the hysteresis increases. This is because as long as the BSN user is predicted to enter another AP with high confidence probability, the handover is performed immediately to reduce outage.

### 4.4. Simulation Results in the Case with Constructive Constraint

**Figure 9.**
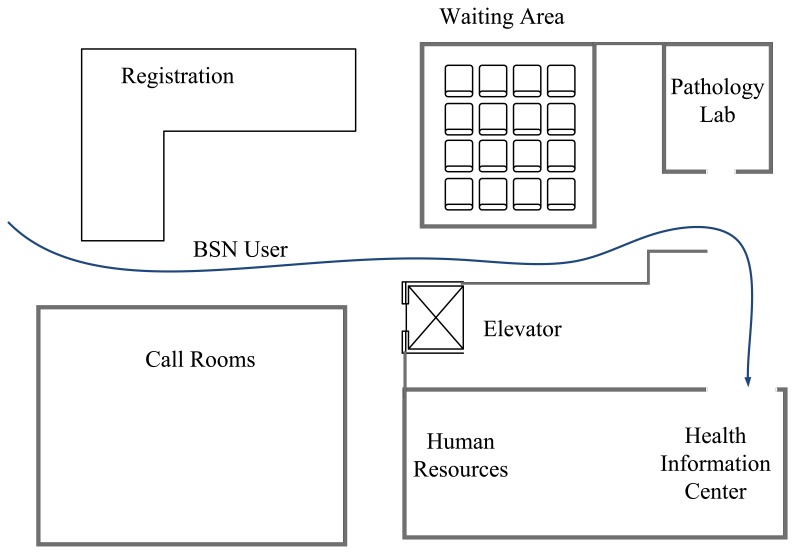
Floor map of Emergency Department in UK Good Samaritan Hospital.

In this subsection, we consider the BSN deployment scenario where BSNs move inside a realistic BSN deployment area with constructive constraints in hospital. Figure 9 shows the floor map of the UK Good Samaritan Hospital Emergency Department area. The standard operational process of emergency is as follows: (1) the patient first registers at the registration counter; (2) the patient then waits for triage at the waiting area; (3) after the triage, the patient goes to the health information center to get preliminary consultations and is later checked in the pathology lab by doctors. When BSNs are deployed in the hospital Emergency Department, BSNs will be operational during processes (2) and (3). One possible route of the BSN user is shown in Figure 9. In the simulation, a BSN user follows certain predefined routes, leaving only the movement direction to be predicted.

**Figure 10.**
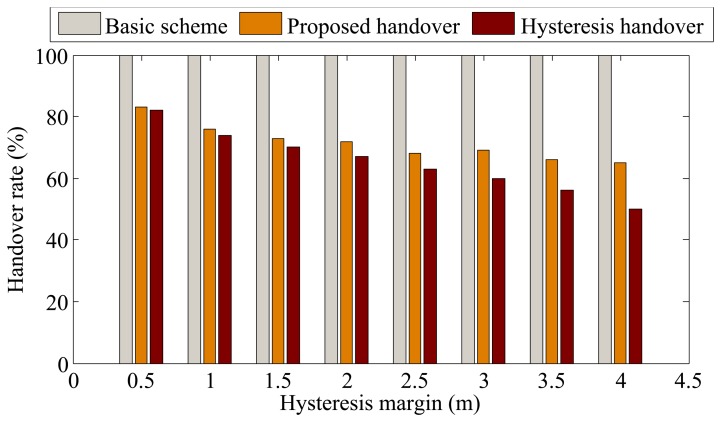
Comparison of handover rate in the BSN deployment case with constructive constraints.

**Figure 11.**
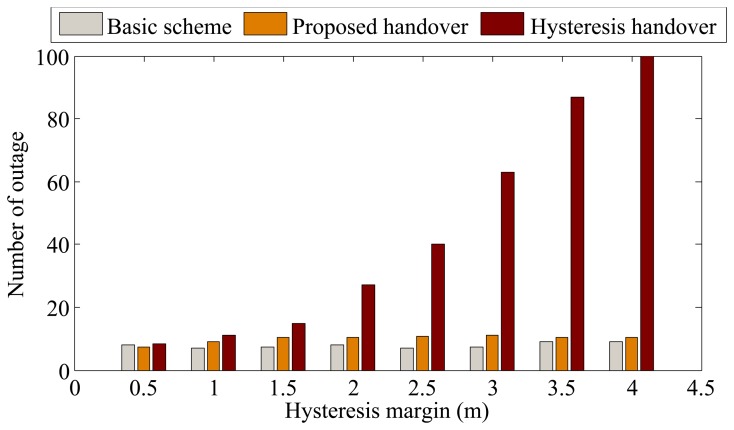
Comparison of the number of outage in the BSN deployment case with constructive constraints.

Figure 10 shows the comparison of handover rate in the BSN deployment scenario with constructive constraints. It can be seem from Figure 10 that the handover rate of the proposed scheme is lower than that of the case without constructive constraints (see Figure 7). This is because when the route of the BSN users is known, the only predicted parameter is movement direction, resulting in more accurate position prediction. Figure 11 shows the number of outages in the case with constructive constraints. For our proposed scheme, the number of outages in Figure 11 is lower than that of the case without constructive constraints (see Figure 8). Similarly, this is because with more accurate position prediction, handover can be performed promptly to avoid outage.

## 5. Conclusions

In this paper, we have proposed an optimized handover scheme with movement trend awareness for body sensor networks (BSNs). The proposed scheme predicts the future position of a BSN user using the movement trend extracted from historical position, and adjusts the handover decision accordingly. Confidence probability is introduced to measure the accuracy of the position prediction and estimate the unnecessary handover rate. Handover initiation time is optimized when the outage probability is minimized and the estimated handover rate meets the requirement. The simulation results showed that the proposed handover scheme reduces the outage probability by 22% as compared with the existing hysteresis-based handover scheme under the constraint of acceptable handover rate. Moreover, when the geometric information of the BSN deployment area is known, the performance of the proposed handover scheme is further improved.

The proposed localization scheme provides an alternate solution to obtain the movement trend of a BSN by taking advantage of the existing inertial sensors of a BSN and the backbone wireless local area network (WLAN). Moreover, the proposed handover scheme can be incorporated into other pedestrian localization schemes, when the localization information of a BSN user can be easily obtained by the BSN coordinator [29–31]. For future work, we will implement the proposed scheme in actual BSN system and evaluate the performance with extensive experiments.

## Figures and Tables

**Table 1.** The notations of the terms.

*v̅*	The average velocity of the historical trajectory
*N*	The number of time index that contribute to *v̅*
*k*	The time index that BSN crosses the boundary
*i*	The time index
*v_i_*	The velocity of BSN at time index *i*
*s_i_*	The position prediction of BSN at time index *i*
*a_i_*	The acceleration of BSN at time index *i*
$\sigma_{a}^{2}$	The variance of acceleration extracted from historical trajectory
*T*	The time interval between two position predictions
$\hat{s_{i}}$	The expectation of *s_i_*
*τ*	The prediction period

**Table 2.** Handover rate and outage times over various localization errors.

**Location Error (m)**	**Handover Rate**	**Drop Call Times**

0.82	0.7532	15
1.21	0.7845	16
1.62	0.7947	15
2.01	0.8062	17
